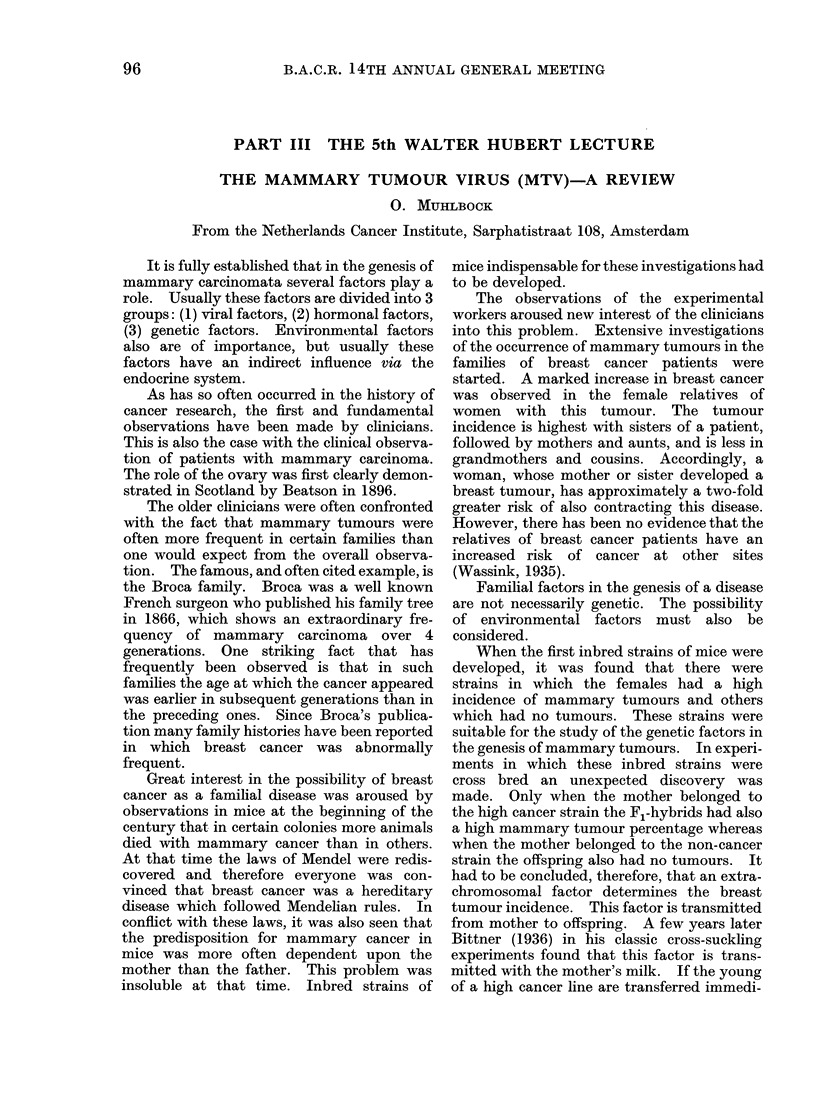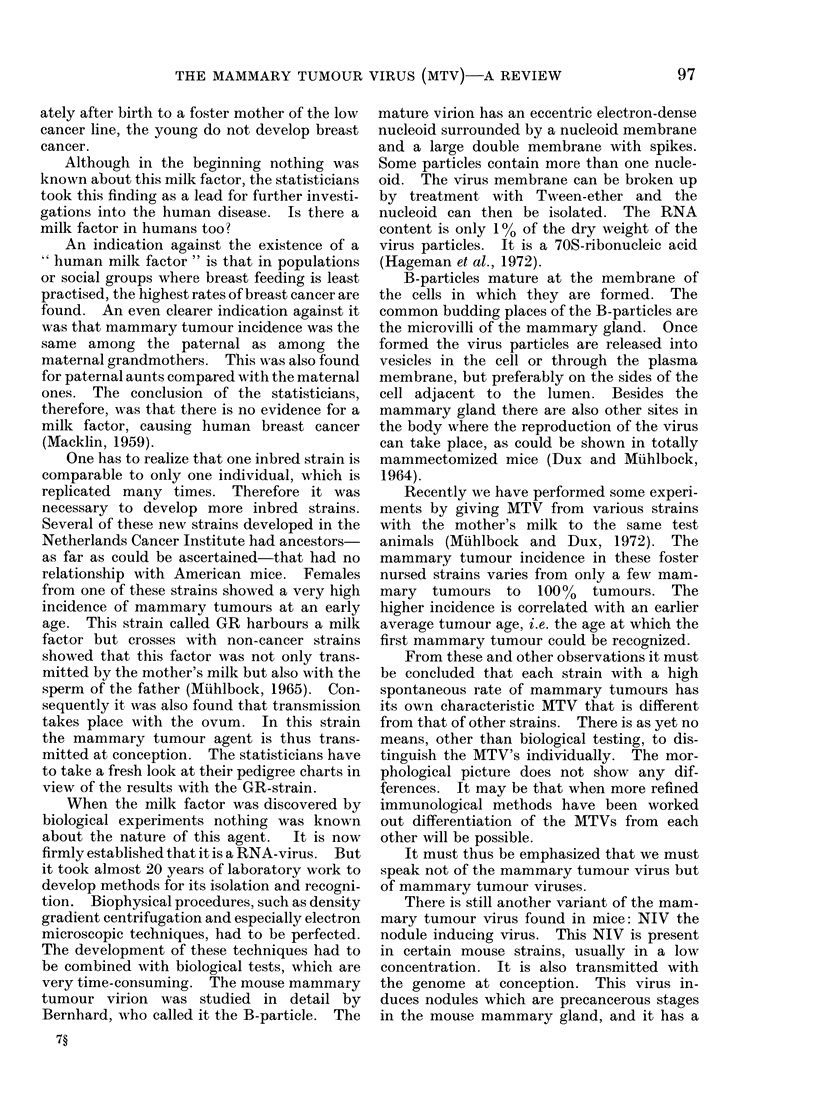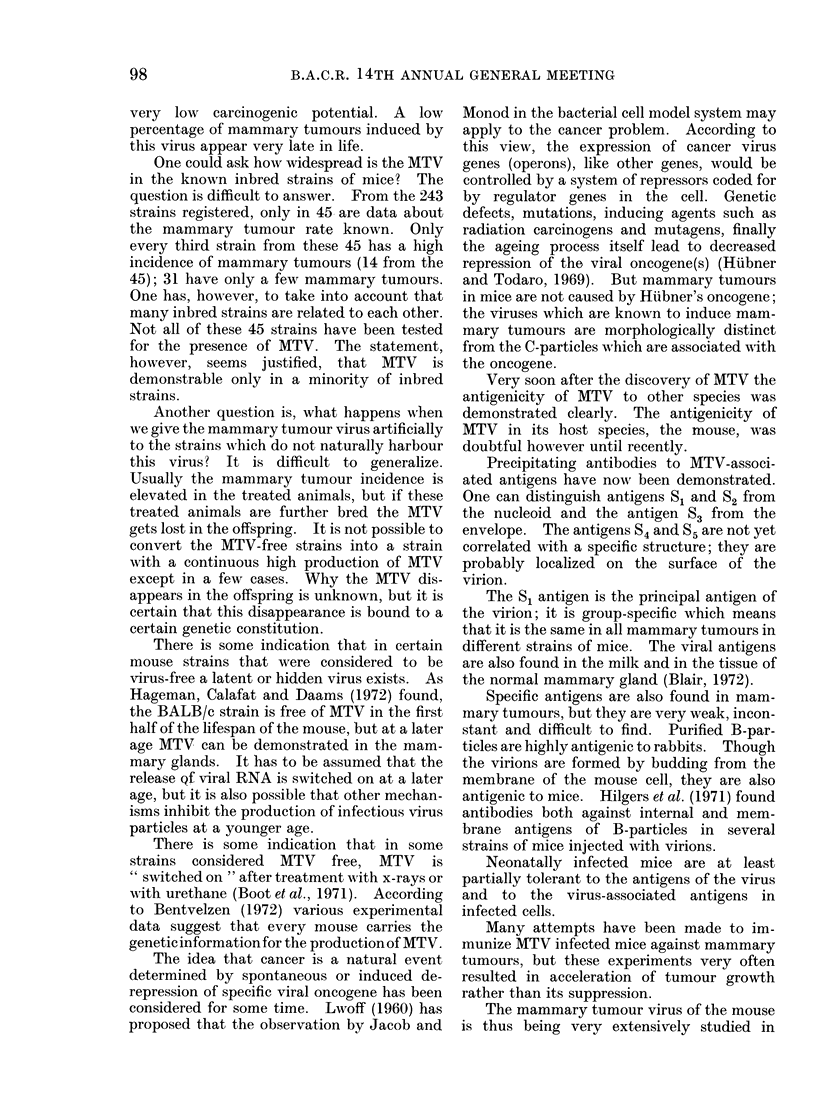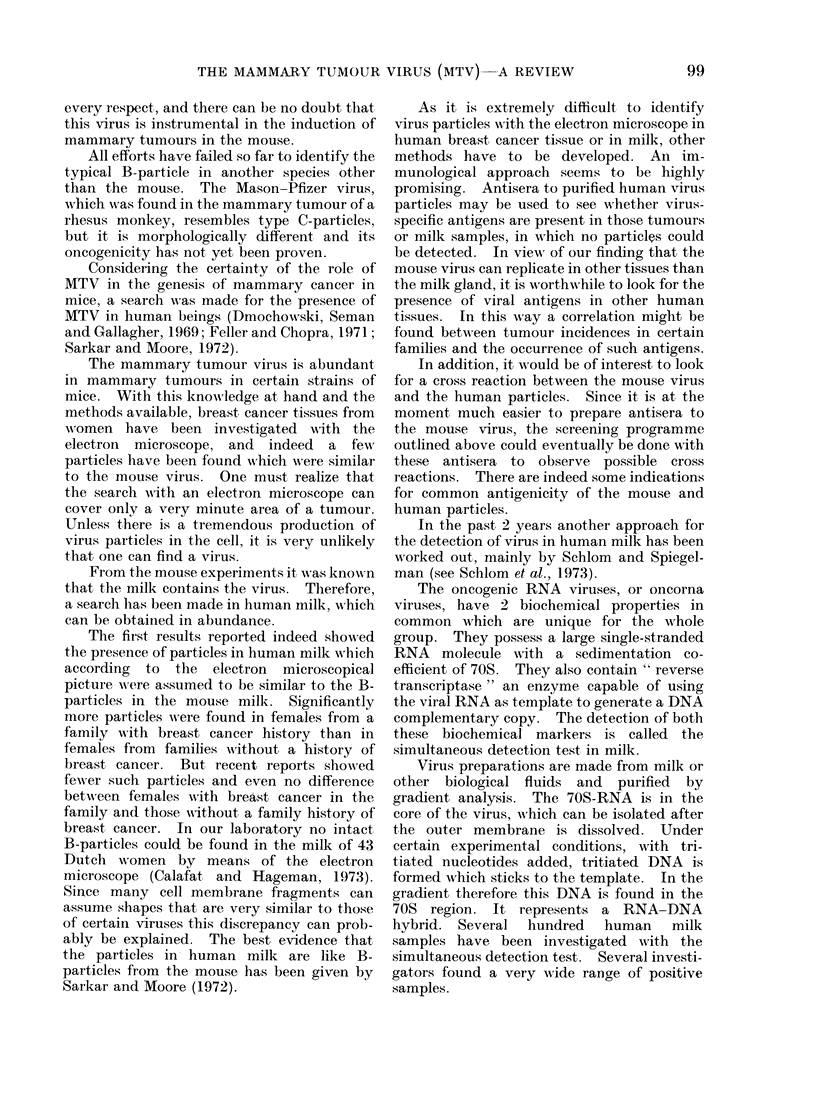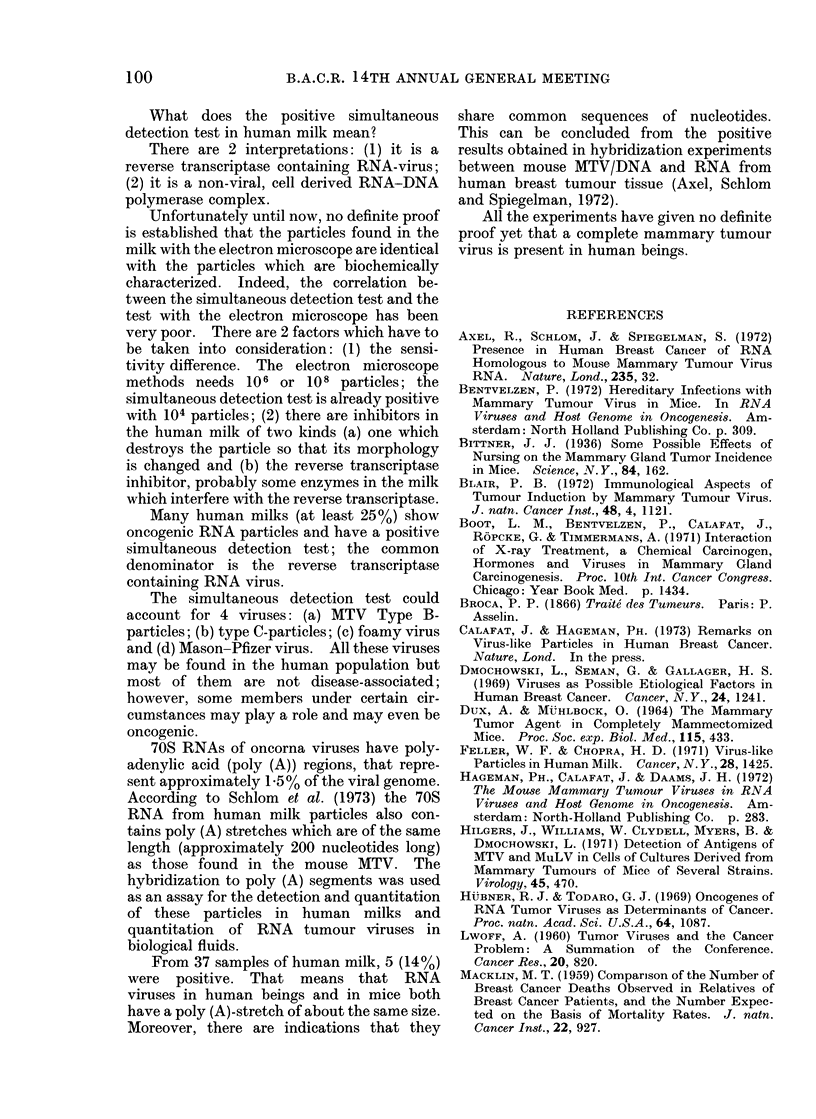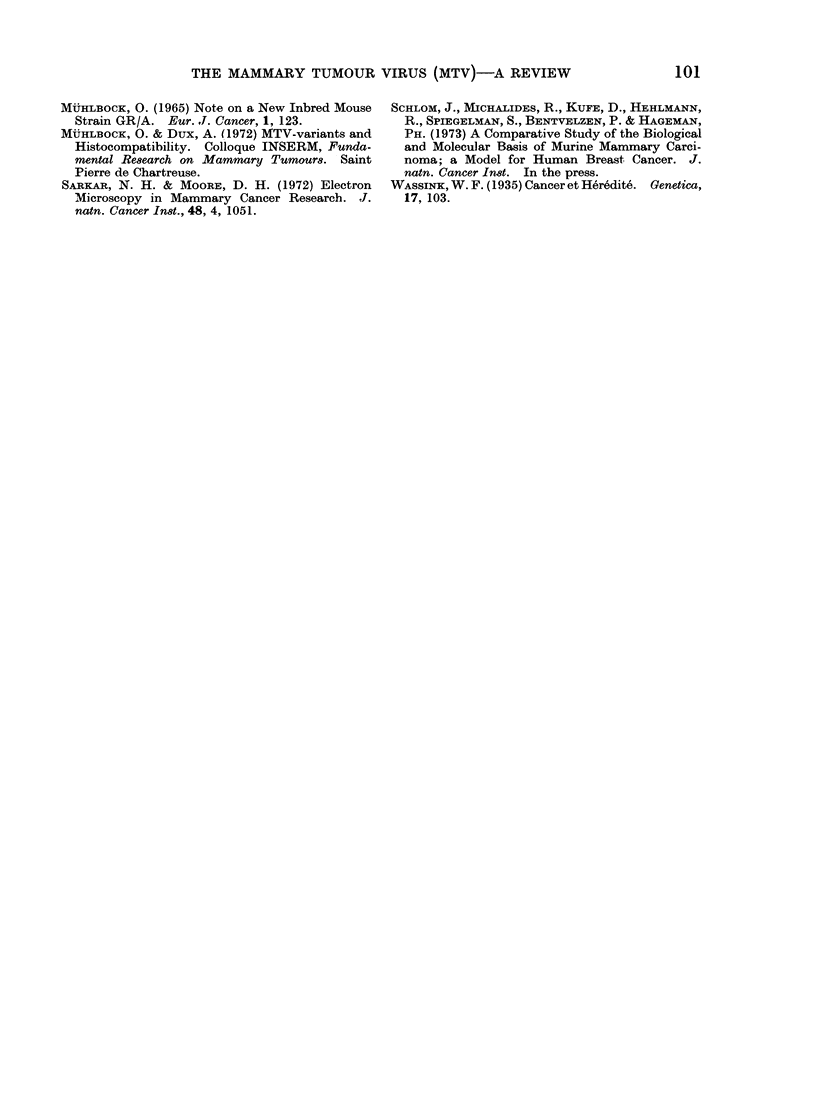# Part III the 5th Walter Hubert Lecture

**Published:** 1973-07

**Authors:** O. Muhlbock


### PART III THE 5th WALTER HUBERT LECTURE

### THE MAMMARY TUMOUR VIRUS (MTV)—A REVIEW

O. MUHLBOCK

From the Netherlands Cancer Institute, Sarphatistraat 108, Amsterdam

It is fully established that in the genesis of mammary carcinomata several factors play a role. Usually these factors are divided into 3 groups: (1) viral factors, (2) hormonal factors, (3) genetic factors. Environmental factors also are of importance, but usually these factors have an indirect influence *via* the endocrine system.

As has so often occurred in the history of cancer research, the first and fundamental observations have been made by clinicians. This is also the case with the clinical observation of patients with mammary carcinoma. The role of the ovary was first clearly demonstrated in Scotland by Beatson in 1896.

The older clinicians were often confronted with the fact that mammary tumours were often more frequent in certain families than one would expect from the overall observation. The famous, and often cited example, is the Broca family. Broca was a well known French surgeon who published his family tree in 1866, which shows an extraordinary frequency of mammary carcinoma over 4 generations. One striking fact that has frequently been observed is that in such families the age at which the cancer appeared was earlier in subsequent generations than in the preceding ones. Since Broca's publication many family histories have been reported in which breast cancer was abnormally frequent.

Great interest in the possibility of breast cancer as a familial disease was aroused by observations in mice at the beginning of the century that in certain colonies more animals died with mammary cancer than in others. At that time the laws of Mendel were rediscovered and therefore everyone was convinced that breast cancer was a hereditary disease which followed Mendelian rules. In conflict with these laws, it was also seen that the predisposition for mammary cancer in mice was more often dependent upon the mother than the father. This problem was insoluble at that time. Inbred strains of mice indispensable for these investigations had to be developed.

The observations of the experimental workers aroused new interest of the clinicians into this problem. Extensive investigations of the occurrence of mammary tumours in the families of breast cancer patients were started. A marked increase in breast cancer was observed in the female relatives of women with this tumour. The tumour incidence is highest with sisters of a patient, followed by mothers and aunts, and is less in grandmothers and cousins. Accordingly, a woman, whose mother or sister developed a breast tumour, has approximately a two-fold greater risk of also contracting this disease. However, there has been no evidence that the relatives of breast cancer patients have an increased risk of cancer at other sites (Wassink, 1935).

Familial factors in the genesis of a disease are not necessarily genetic. The possibility of environmental factors must also be considered.

When the first inbred strains of mice were developed, it was found that there were strains in which the females had a high incidence of mammary tumours and others which had no tumours. These strains were suitable for the study of the genetic factors in the genesis of mammary tumours. In experiments in which these inbred strains were cross bred an unexpected discovery was made. Only when the mother belonged to the high cancer strain the $F_1$-hybrids had also a high mammary tumour percentage whereas when the mother belonged to the non-cancer strain the offspring also had no tumours. It had to be concluded, therefore, that an extra-chromosomal factor determines the breast tumour incidence. This factor is transmitted from mother to offspring. A few years later Bittner (1936) in his classic cross-suckling experiments found that this factor is transmitted with the mother's milk. If the young of a high cancer line are transferred immediately after birth to a foster mother of the low cancer line, the young do not develop breast cancer.

Although in the beginning nothing was known about this milk factor, the statisticians took this finding as a lead for further investigations into the human disease. Is there a milk factor in humans too?

An indication against the existence of a " human milk factor " is that in populations or social groups where breast feeding is least practised, the highest rates of breast cancer are found. An even clearer indication against it was that mammary tumour incidence was the same among the paternal as among the maternal grandmothers. This was also found for paternal aunts compared with the maternal ones. The conclusion of the statisticians, therefore, was that there is no evidence for a milk factor, causing human breast cancer (Macklin, 1959).

One has to realize that one inbred strain is comparable to only one individual, which is replicated many times. Therefore it was necessary to develop more inbred strains. Several of these new strains developed in the Netherlands Cancer Institute had ancestors—as far as could be ascertained—that had no relationship with American mice. Females from one of these strains showed a very high incidence of mammary tumours at an early age. This strain called GR harbours a milk factor but crosses with non-cancer strains showed that this factor was not only transmitted by the mother's milk but also with the sperm of the father (Mühlbock, 1965). Consequently it was also found that transmission takes place with the ovum. In this strain the mammary tumour agent is thus transmitted at conception. The statisticians have to take a fresh look at their pedigree charts in view of the results with the GR-strain.

When the milk factor was discovered by biological experiments nothing was known about the nature of this agent. It is now firmly established that it is a RNA-virus. But it took almost 20 years of laboratory work to develop methods for its isolation and recognition. Biophysical procedures, such as density gradient centrifugation and especially electron microscopic techniques, had to be perfected. The development of these techniques had to be combined with biological tests, which are very time-consuming. The mouse mammary tumour virion was studied in detail by Bernhard, who called it the B-particle. The mature virion has an eccentric electron-dense nucleoid surrounded by a nucleoid membrane and a large double membrane with spikes. Some particles contain more than one nucleoid. The virus membrane can be broken up by treatment with Tween-ether and the nucleoid can then be isolated. The RNA content is only 1% of the dry weight of the virus particles. It is a 70S-ribonucleic acid (Hageman *et al.*, 1972).

B-particles mature at the membrane of the cells in which they are formed. The common budding places of the B-particles are the microvilli of the mammary gland. Once formed the virus particles are released into vesicles in the cell or through the plasma membrane, but preferably on the sides of the cell adjacent to the lumen. Besides the mammary gland there are also other sites in the body where the reproduction of the virus can take place, as could be shown in totally mammectomized mice (Dux and Mühlbock, 1964).

Recently we have performed some experiments by giving MTV from various strains with the mother's milk to the same test animals (Mühlbock and Dux, 1972). The mammary tumour incidence in these foster nursed strains varies from only a few mammary tumours to 100% tumours. The higher incidence is correlated with an earlier average tumour age, *i.e.* the age at which the first mammary tumour could be recognized.

From these and other observations it must be concluded that each strain with a high spontaneous rate of mammary tumours has its own characteristic MTV that is different from that of other strains. There is as yet no means, other than biological testing, to distinguish the MTV's individually. The morphological picture does not show any differences. It may be that when more refined immunological methods have been worked out differentiation of the MTVs from each other will be possible.

It must thus be emphasized that we must speak not of the mammary tumour virus but of mammary tumour viruses.

There is still another variant of the mammary tumour virus found in mice: NIV the nodule inducing virus. This NIV is present in certain mouse strains, usually in a low concentration. It is also transmitted with the genome at conception. This virus induces nodules which are precancerous stages in the mouse mammary gland, and it has a very low carcinogenic potential. A low percentage of mammary tumours induced by this virus appear very late in life.

One could ask how widespread is the MTV in the known inbred strains of mice? The question is difficult to answer. From the 243 strains registered, only in 45 are data about the mammary tumour rate known. Only every third strain from these 45 has a high incidence of mammary tumours (14 from the 45); 31 have only a few mammary tumours. One has, however, to take into account that many inbred strains are related to each other. Not all of these 45 strains have been tested for the presence of MTV. The statement, however, seems justified, that MTV is demonstrable only in a minority of inbred strains.

Another question is, what happens when we give the mammary tumour virus artificially to the strains which do not naturally harbour this virus? It is difficult to generalize. Usually the mammary tumour incidence is elevated in the treated animals, but if these treated animals are further bred the MTV gets lost in the offspring. It is not possible to convert the MTV-free strains into a strain with a continuous high production of MTV except in a few cases. Why the MTV disappears in the offspring is unknown, but it is certain that this disappearance is bound to a certain genetic constitution.

There is some indication that in certain mouse strains that were considered to be virus-free a latent or hidden virus exists. As Hageman, Calafat and Daams (1972) found, the BALB/c strain is free of MTV in the first half of the lifespan of the mouse, but at a later age MTV can be demonstrated in the mammary glands. It has to be assumed that the release of viral RNA is switched on at a later age, but it is also possible that other mechanisms inhibit the production of infectious virus particles at a younger age.

There is some indication that in some strains considered MTV free, MTV is " switched on " after treatment with x-rays or with urethane (Boot *et al.*, 1971). According to Bentvelzen (1972) various experimental data suggest that every mouse carries the genetic information for the production of MTV.

The idea that cancer is a natural event determined by spontaneous or induced derepression of specific viral oncogene has been considered for some time. Lwoff (1960) has proposed that the observation by Jacob and Monod in the bacterial cell model system may apply to the cancer problem. According to this view, the expression of cancer virus genes (operons), like other genes, would be controlled by a system of repressors coded for by regulator genes in the cell. Genetic defects, mutations, inducing agents such as radiation carcinogens and mutagens, finally the ageing process itself lead to decreased repression of the viral oncogene(s) (Hübner and Todaro, 1969). But mammary tumours in mice are not caused by Hübner's oncogene; the viruses which are known to induce mammary tumours are morphologically distinct from the C-particles which are associated with the oncogene.

Very soon after the discovery of MTV the antigenicity of MTV to other species was demonstrated clearly. The antigenicity of MTV in its host species, the mouse, was doubtful however until recently.

Precipitating antibodies to MTV-associated antigens have now been demonstrated. One can distinguish antigens $S_1$ and $S_2$ from the nucleoid and the antigen $S_3$ from the envelope. The antigens $S_4$ and $S_5$ are not yet correlated with a specific structure; they are probably localized on the surface of the virion.

The $S_1$ antigen is the principal antigen of the virion; it is group-specific which means that it is the same in all mammary tumours in different strains of mice. The viral antigens are also found in the milk and in the tissue of the normal mammary gland (Blair, 1972).

Specific antigens are also found in mammary tumours, but they are very weak, inconstant and difficult to find. Purified B-particles are highly antigenic to rabbits. Though the virions are formed by budding from the membrane of the mouse cell, they are also antigenic to mice. Hilgers *et al.* (1971) found antibodies both against internal and membrane antigens of B-particles in several strains of mice injected with virions.

Neonatally infected mice are at least partially tolerant to the antigens of the virus and to the virus-associated antigens in infected cells.

Many attempts have been made to immunize MTV infected mice against mammary tumours, but these experiments very often resulted in acceleration of tumour growth rather than its suppression.

The mammary tumour virus of the mouse is thus being very extensively studied in every respect, and there can be no doubt that this virus is instrumental in the induction of mammary tumours in the mouse.

All efforts have failed so far to identify the typical B-particle in another species other than the mouse. The Mason–Pfizer virus, which was found in the mammary tumour of a rhesus monkey, resembles type C-particles, but it is morphologically different and its oncogenicity has not yet been proven.

Considering the certainty of the role of MTV in the genesis of mammary cancer in mice, a search was made for the presence of MTV in human beings (Dmochowski, Seman and Gallagher, 1969; Feller and Chopra, 1971; Sarkar and Moore, 1972).

The mammary tumour virus is abundant in mammary tumours in certain strains of mice. With this knowledge at hand and the methods available, breast cancer tissues from women have been investigated with the electron microscope, and indeed a few particles have been found which were similar to the mouse virus. One must realize that the search with an electron microscope can cover only a very minute area of a tumour. Unless there is a tremendous production of virus particles in the cell, it is very unlikely that one can find a virus.

From the mouse experiments it was known that the milk contains the virus. Therefore, a search has been made in human milk, which can be obtained in abundance.

The first results reported indeed showed the presence of particles in human milk which according to the electron microscopical picture were assumed to be similar to the B-particles in the mouse milk. Significantly more particles were found in females from a family with breast cancer history than in females from families without a history of breast cancer. But recent reports showed fewer such particles and even no difference between females with breast cancer in the family and those without a family history of breast cancer. In our laboratory no intact B-particles could be found in the milk of 43 Dutch women by means of the electron microscope (Calafat and Hageman, 1973). Since many cell membrane fragments can assume shapes that are very similar to those of certain viruses this discrepancy can probably be explained. The best evidence that the particles in human milk are like B-particles from the mouse has been given by Sarkar and Moore (1972).

As it is extremely difficult to identify virus particles with the electron microscope in human breast cancer tissue or in milk, other methods have to be developed. An immunological approach seems to be highly promising. Antisera to purified human virus particles may be used to see whether virus-specific antigens are present in those tumours or milk samples, in which no particles could be detected. In view of our finding that the mouse virus can replicate in other tissues than the milk gland, it is worthwhile to look for the presence of viral antigens in other human tissues. In this way a correlation might be found between tumour incidences in certain families and the occurrence of such antigens.

In addition, it would be of interest to look for a cross reaction between the mouse virus and the human particles. Since it is at the moment much easier to prepare antisera to the mouse virus, the screening programme outlined above could eventually be done with these antisera to observe possible cross reactions. There are indeed some indications for common antigenicity of the mouse and human particles.

In the past 2 years another approach for the detection of virus in human milk has been worked out, mainly by Schlom and Spiegelman (see Schlom *et al.*, 1973).

The oncogenic RNA viruses, or oncorna viruses, have 2 biochemical properties in common which are unique for the whole group. They possess a large single-stranded RNA molecule with a sedimentation coefficient of 70S. They also contain " reverse transcriptase " an enzyme capable of using the viral RNA as template to generate a DNA complementary copy. The detection of both these biochemical markers is called the simultaneous detection test in milk.

Virus preparations are made from milk or other biological fluids and purified by gradient analysis. The 70S-RNA is in the core of the virus, which can be isolated after the outer membrane is dissolved. Under certain experimental conditions, with tritiated nucleotides added, tritiated DNA is formed which sticks to the template. In the gradient therefore this DNA is found in the 70S region. It represents a RNA–DNA hybrid. Several hundred human milk samples have been investigated with the simultaneous detection test. Several investigators found a very wide range of positive samples.

What does the positive simultaneous detection test in human milk mean?

There are 2 interpretations: (1) it is a reverse transcriptase containing RNA-virus; (2) it is a non-viral, cell derived RNA–DNA polymerase complex.

Unfortunately until now, no definite proof is established that the particles found in the milk with the electron microscope are identical with the particles which are biochemically characterized. Indeed, the correlation between the simultaneous detection test and the test with the electron microscope has been very poor. There are 2 factors which have to be taken into consideration: (1) the sensitivity difference. The electron microscope methods needs $10^6$ or $10^8$ particles; the simultaneous detection test is already positive with $10^4$ particles; (2) there are inhibitors in the human milk of two kinds (a) one which destroys the particle so that its morphology is changed and (b) the reverse transcriptase inhibitor, probably some enzymes in the milk which interfere with the reverse transcriptase.

Many human milks (at least 25%) show oncogenic RNA particles and have a positive simultaneous detection test; the common denominator is the reverse transcriptase containing RNA virus.

The simultaneous detection test could account for 4 viruses: (a) MTV Type B-particles; (b) type C-particles; (c) foamy virus and (d) Mason–Pfizer virus. All these viruses may be found in the human population but most of them are not disease-associated; however, some members under certain circumstances may play a role and may even be oncogenic.

70S RNAs of oncorna viruses have poly-adenylic acid (poly (A)) regions, that represent approximately 1·5% of the viral genome. According to Schlom *et al.* (1973) the 70S RNA from human milk particles also contains poly (A) stretches which are of the same length (approximately 200 nucleotides long) as those found in the mouse MTV. The hybridization to poly (A) segments was used as an assay for the detection and quantitation of these particles in human milks and quantitation of RNA tumour viruses in biological fluids.

From 37 samples of human milk, 5 (14%) were positive. That means that RNA viruses in human beings and in mice both have a poly (A)-stretch of about the same size. Moreover, there are indications that they share common sequences of nucleotides. This can be concluded from the positive results obtained in hybridization experiments between mouse MTV/DNA and RNA from human breast tumour tissue (Axel, Schlom and Spiegelman, 1972).

All the experiments have given no definite proof yet that a complete mammary tumour virus is present in human beings.

### REFERENCES

Axel, R., Schlom, J. & Spiegelman, S. (1972) Presence in Human Breast Cancer of RNA Homologous to Mouse Mammary Tumour Virus RNA. *Nature, Lond.*, **235**, 32.

Bentvelzen, P. (1972) Hereditary Infections with Mammary Tumour Virus in Mice. In *RNA Viruses and Host Genome in Oncogenesis.* Amsterdam: North Holland Publishing Co. p. 309.

Bittner, J. J. (1936) Some Possible Effects of Nursing on the Mammary Gland Tumor Incidence in Mice. *Science, N.Y.*, **84**, 162.

Blair, P. B. (1972) Immunological Aspects of Tumour Induction by Mammary Tumour Virus. *J. natn. Cancer Inst.*, **48**, 4, 1121.

Boot, L. M., Bentvelzen, P., Calafat, J., Röpcke, G. & Timmermans, A. (1971) Interaction of X-ray Treatment, a Chemical Carcinogen, Hormones and Viruses in Mammary Gland Carcinogenesis. *Proc. 10th Int. Cancer Congress.* Chicago: Year Book Med. p. 1434.

Broca, P. P. (1866) *Traité des Tumeurs.* Paris: P. Asselin.

Calafat, J. & Hageman, Ph. (1973) Remarks on Virus-like Particles in Human Breast Cancer. *Nature, Lond.* In the press.

Dmochowski, L., Seman, G. & Gallager, H. S. (1969) Viruses as Possible Etiological Factors in Human Breast Cancer. *Cancer, N.Y.*, **24**, 1241.

Dux, A. & Mühlbock, O. (1964) The Mammary Tumor Agent in Completely Mammectomized Mice. *Proc. Soc. exp. Biol. Med.*, **115**, 433.

Feller, W. F. & Chopra, H. D. (1971) Virus-like Particles in Human Milk. *Cancer, N.Y.*, **28**, 1425.

Hageman, Ph., Calafat, J. & Daams, J. H. (1972) *The Mouse Mammary Tumour Viruses in RNA Viruses and Host Genome in Oncogenesis.* Amsterdam: North-Holland Publishing Co. p. 283.

Hilgers, J., Williams, W. Clydell, Myers, B. & Dmochowski, L. (1971) Detection of Antigens of MTV and MuLV in Cells of Cultures Derived from Mammary Tumours of Mice of Several Strains. *Virology*, **45**, 470.

Hübner, R. J. & Todaro, G. J. (1969) Oncogenes of RNA Tumor Viruses as Determinants of Cancer. *Proc. natn. Acad. Sci. U.S.A.*, **64**, 1087.

Lwoff, A. (1960) Tumor Viruses and the Cancer Problem: A Summation of the Conference. *Cancer Res.*, **20**, 820.

Macklin, M. T. (1959) Comparison of the Number of Breast Cancer Deaths Observed in Relatives of Breast Cancer Patients, and the Number Expected on the Basis of Mortality Rates. *J. natn. Cancer Inst.*, **22**, 927.

Mühlbock, O. (1965) Note on a New Inbred Mouse Strain GR/A. *Eur. J. Cancer*, **1**, 123.

Mühlbock, O. & Dux, A. (1972) MTV-variants and Histocompatibility. Colloque INSERM, *Fundamental Research on Mammary Tumours.* Saint Pierre de Chartreuse.

Sarkar, N. H. & Moore, D. H. (1972) Electron Microscopy in Mammary Cancer Research. *J. natn. Cancer Inst.*, **48**, 4, 1051.

Schlom, J., Michalides, R., Kufe, D., Hehlmann, R., Spiegelman, S., Bentvelzen, P. & Hageman, Ph. (1973) A Comparative Study of the Biological and Molecular Basis of Murine Mammary Carcinoma; a Model for Human Breast Cancer. *J. natn. Cancer Inst.* In the press.

Wassink, W. F. (1935) Cancer et Hérédité. *Genetica*, **17**, 103.